# Selective Sorting of Semiconducting C_70_@Single‐Walled Carbon Nanotube Heterostructures with Narrow Diameter Distribution

**DOI:** 10.1002/advs.202500933

**Published:** 2025-03-16

**Authors:** Yuqi He, Jian Yao, Ye Liu, Feng Jin, Yujie Peng, Zeyuan Nan, Aling Chen, Hehua Jin, Song Qiu, Lixing Kang, Dengsong Zhang, Qingwen Li

**Affiliations:** ^1^ Department of Chemistry College of Sciences Shanghai University Shanghai 200444 P. R. China; ^2^ Advanced Materials Division Suzhou Institute of Nano‐Tech and Nano‐Bionics Chinese Academy of Sciences Suzhou 215123 P. R. China; ^3^ School of Nano‐Technology and Nano‐Bionics University of Science and Technology of China Hefei 230026 P. R. China

**Keywords:** heterostructure, single‐walled carbon nanotube, encapsulation, fullerene, selective sorting, semiconductor

## Abstract

Consistently arranging molecules within single‐walled carbon nanotube (SWCNT) templates shows promise for creating advanced 1D heterostructures, but diameter variations in raw SWCNTs pose a significant challenge. In this work, a precise synthesis of C_70_ fullerene‐filled SWCNTs (C_70_@SWCNTs) is achieved through vapor‐phase filling followed by polymer sorting. As the SWCNT diameter increases, C_70_ molecules first stack in a single chain, then form unusual configurations, including staggered double chains and double helices—configurations not observed in bulk C_70_ crystals. SWCNT deformation, which is often overlooked in previous theoretical works, is found to significantly alter the C_70_ stacking configuration. C_70_‐SWCNT electronic interactions, particularly charge transfer, allow selective extraction of C_70_@SWCNTs with narrowly distributed diameters and good semiconducting purity. The sorted C_70_@SWCNTs have diameters of 1.3–1.4 nm, corresponding to C_70_‐SWCNT distances of ca. 0.34 nm, where the strongest electronic interactions occur. An on/off current ratio of 10⁴ is achieved in their field‐effect transistors. The synthesis and separation strategy sheds light on the preparation and application of 1D heterostructures.

## Graphical abstract

A precise synthesis of C_70_ fullerene‐filled SWCNTs (C_70_@SWCNTs) is achieved through vapor‐phase filling followed by polymer sorting. As the SWCNT diameter increases, C_70_ molecules first stack in a single chain, and then form unusual configurations, including staggered double chains and double helices. C_70_‐SWCNT electronic interactions, particularly charge transfer, allow us to selectively extract C_70_@SWCNTs with narrowly distributed diameters and good semiconducting purity.

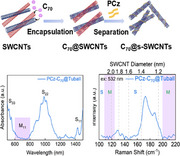

## 1 Introduction

The nanosized cavities of single‐walled carbon nanotubes (SWCNTs), constructed by thermally and chemically stable walls, provide an ideal platform for synthesizing 1D structures through encapsulation of molecules.^[^ ^1^, ^2^, ^3^ ^]^ Researchers have encapsulated fullerenes,^[^ ^4^, ^5^, ^6^, ^7^ ^]^ clusters,^[^ ^8^, ^9^ ^]^ organic molecules,^[^ ^10^, ^11^, ^12^, ^13^, ^14^, ^15^ ^]^ and one‐dimensional elements^[^ ^16^, ^17^ ^]^ or alloys^[^ ^18^ ^]^ within SWCNTs. Encapsulated materials can modulate the electronic,^[^ ^10^, ^12^, ^19^, ^20^, ^21^ ^]^ optical,^[^ ^13^, ^15^, ^22^ ^]^ or thermal^[^ ^23^ ^]^ properties of the SWCNTs, or impart new functionalities, such as magnetism,^[^ ^7^, ^12^ ^]^ making encapsulated SWCNTs promising candidates for applications in nanoelectronics, energy conversion, and catalysis.

One of the first studies on filled SWCNTs refers to the observation of C_60_ molecules in‐situ formed inside SWCNTs in 1998,^[^ ^4^ ^]^ followed by intentional filling of SWCNTs with fullerenes (e.g., C_60_ and C_70_) and their derivatives (e.g., endohedral metallofullerenes).^[^ ^7^, ^19^, ^23^, ^24^, ^25^ ^]^ It is found that filled fullerenes can alter the electronic and optical band gaps,^[^ ^19^, ^20^ ^]^ inner dielectric environment^[^ ^24^ ^]^ and acoustic phonon mode^[^ ^23^ ^]^ of SWCNTs, all of which require further investigation. Especially for C_70_, due to its strong light absorption characteristics and efficient electron transport capabilities, it is a key material in the field of optical and electrical devices.^[^ ^26^, ^27^ ^]^ Elucidating the electrical and spatial interactions within the SWCNT cavities demands high‐precision synthesis and high‐resolution visibility on a sub‐nanometer scale. However, although recent advancements in transmission electron microscopy (TEM) technology have enabled atomic‐level resolution, precise synthesis remains challenging, primarily due to the uncontrolled diameter and chirality distributions of SWCNTs in raw materials. Previous simulations and experiments have reported that changes in SWCNTs diameter can result in diverse fullerene ordering configurations, such as single, multiple chains, or helices, with varied fullerene‐SWCNTs and fullerene‐fullerene distances in each arrangement.^[^ ^5^, ^25^, ^28^, ^29^ ^]^ Moreover, apart from the unclear fullerene arrangements, the mixture of semiconducting SWCNTs (s‐SWCNTs) and metallic SWCNTs (m‐SWCNTs) in raw material also limits semiconductor applications of filling SWCNTs.

We can employ solution‐processed methods to separate SWCNTs after encapsulation, which serves as a strategy for obtaining filled SWCNTs with the desired structures and performance characteristics. So far, selective sorting of unfilled SWCNTs according to their metallicity, diameter, and chirality has been achieved by many solution‐processed methods,^[^ ^30^, ^31^, ^32^, ^33^, ^34^, ^35^, ^36^, ^37^ ^]^ especially the conjugated polymer extraction (CPE) method characterized by high sorting yield and purity.^[^ ^38^, ^39^ ^]^ In the scarce reports of sorting of filled SWCNTs, host‐guest interactions can change the dispersion status of SWCNTs, therefore leading to unattainable sorting results compared with unfilled SWCNTs. Yang et al. isolate large‐diameter semiconducting and metallic SWCNTs with the help of filled polyoxometalate clusters and molybdenum carbide, respectively.^[^ ^9^, ^40^ ^]^ The charge redistribution between SWCNTs and the filled materials is critical for achieving the sorting outcomes.^[^ ^9^ ^]^ In a report from Li et al., alkane‐filling contributes to a rarely observed enantiomer‐level isolation of SWCNTs with large‐diameter.^[^ ^33^ ^]^ Considering the diverse configurations of encapsulated fullerenes, sorting of fullerene‐filled SWCNTs remains an active area to be explored.

In this work, we achieved a precise preparation of C_70_ filled SWCNTs (C_70_@SWCNTs) by combining vapor‐phase sublimation filling with after‐synthesis CPE sorting. With the use of an aberration corrected transmission electron microscope (AC‐TEM), we observed that, with the SWCNT diameter expanding from ca. 1.3 to ca. 2.0 nm, the encapsulated C_70_ assemblies transformed from single to double chains. Moreover, in SWCNTs with ca.1.6 nm diameter, the encapsulated C_70_ can form a double‐helix structure which was unknown in the bulk forms of C_70_. The C_70_ helix structure showed rotational periodicities much longer than the previous theory prediction due to significant wall deformation of SWCNTs. Benefiting from C_70_‐SWCNT electronic interactions, particularly charge transfer we can selectively extract C_70_@SWCNTs with narrowly distributed diameters and high semiconducting purity. The charge redistribution effect between C_70_ and SWCNTs was revealed by Raman and photoelectron spectroscopy characterizations and DFT calculation. The sorted C_70_@SWCNTs have diameters of 1.3–1.4 nm, corresponding to C_70_‐SWCNT distances of ca. 0.34 nm where the strongest electronic interactions occur. Finally, we fabricated field effect transistors based on the sorted C_70_@SWCNTs, and the devices demonstrate the on/off ratio of current ca. 10^4^.

## 2 Results and Discussion

### 2.1 Encapsulation and Structural Characterizations of C_70_@SWCNTs

**Figure 1:**
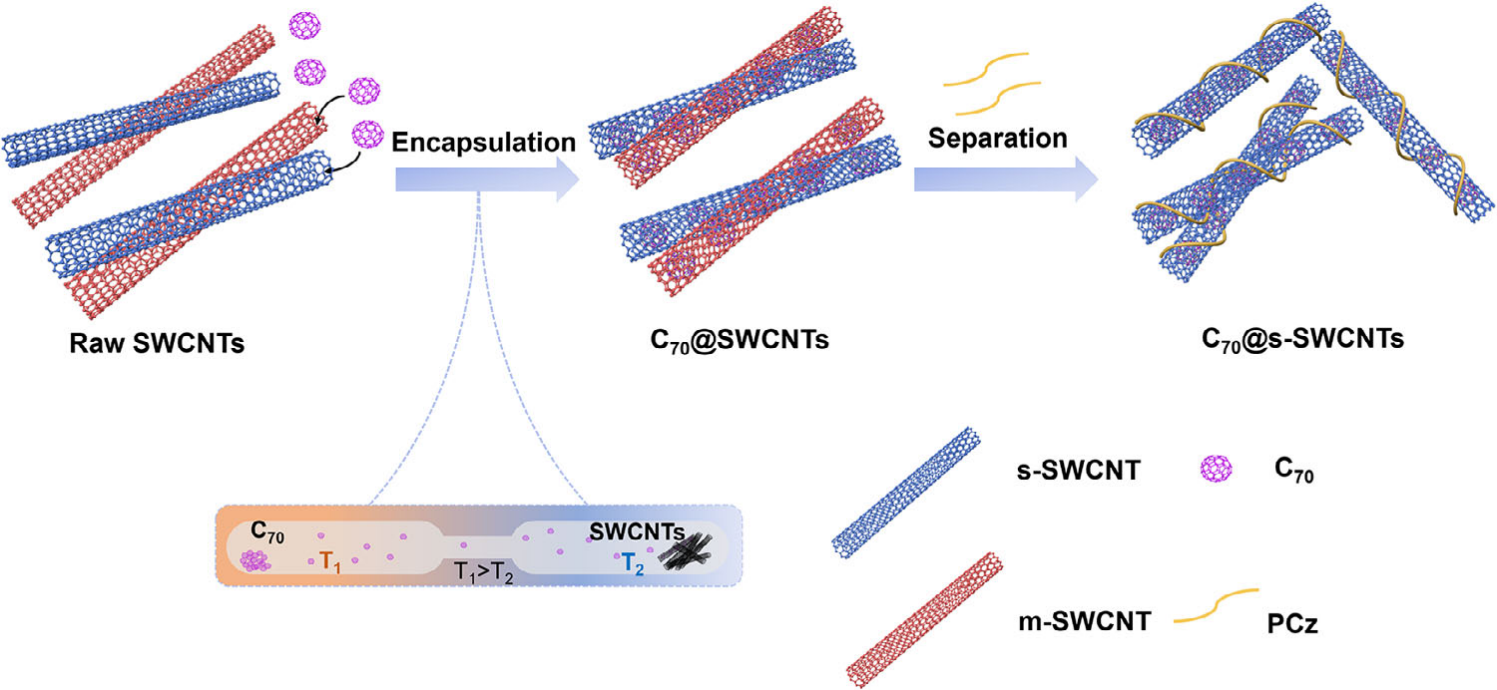
Scheme for C_70_ encapsulation in SWCNTs and semiconducting sorting of C_70_@SWCNTs using conjugated polymer extraction.

As illustrated in **Figure** **1**, C_70_@SWCNTs were prepared using a vapor‐phase sublimation filling method, and were sorted using conjugated polymer extraction. In our group's previous work, we achieved the filling of other materials by the vapor‐phase sublimation filling method.^[^ ^21^, ^41^ ^]^ The Poly[9‐(1‐octanoyl)‐9H‐carbazole‐2,7‐diyl] (PCz) polymer was selected for the separation of C_70_@SWCNTs based on our group's previous high‐purity s‐SWCNTs separation results.^[^ ^42^ ^]^

**Figure 2:**
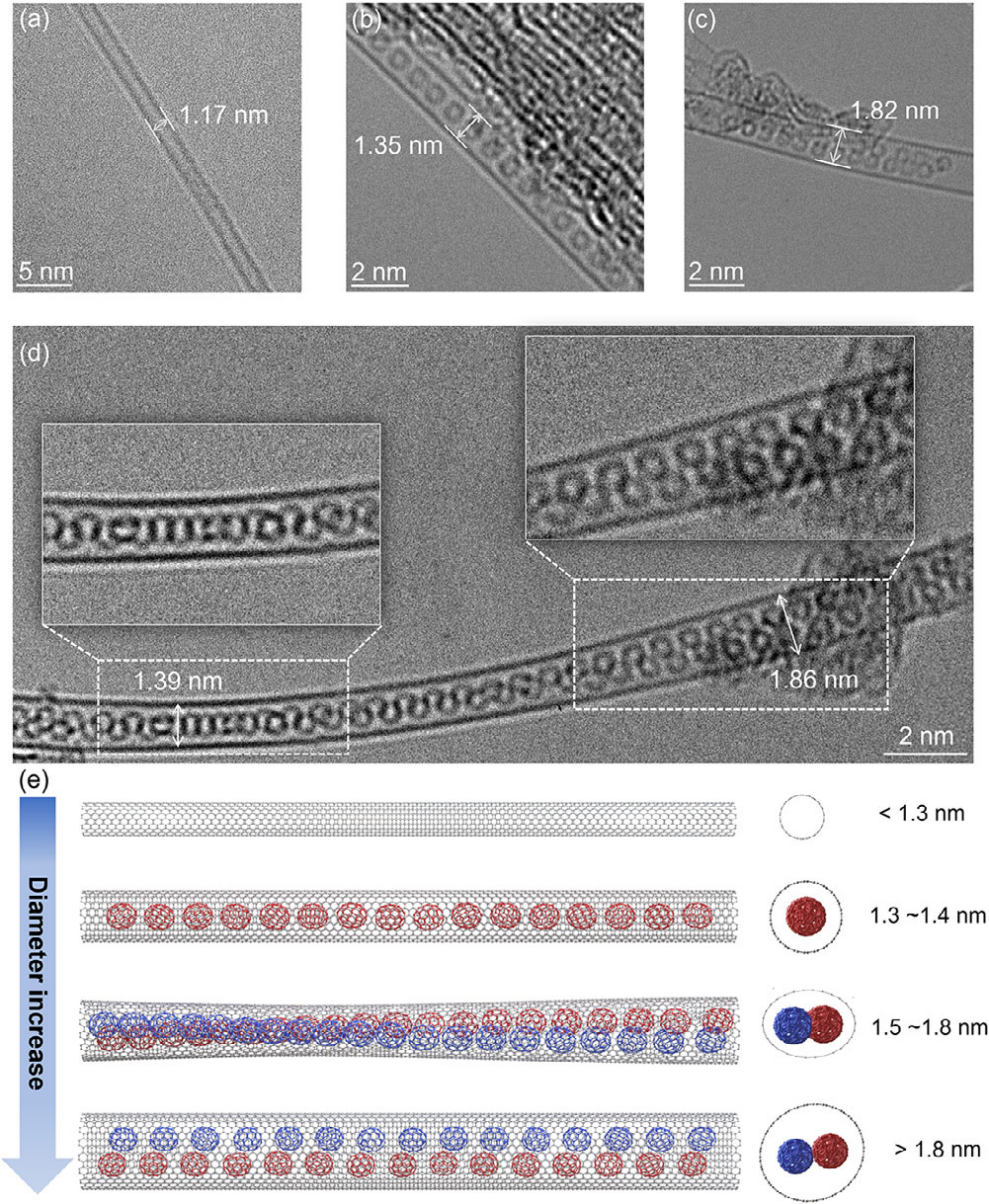
Different ordering configurations of encapsulated C_70_ depending on SWCNT diameters. Representative AC‐TEM images of a) unfilled SWCNTs and filled SWCNTs with b) single chain c) double chains and d) double helix structure of C_70_. e) Structure models of unfilled and C_70_ filled SWCNTs of different diameters.

In the C_70_ encapsulation experiments, we tested Tuball‐SWCNTs with broad‐distributed diameters (1.2–2 nm). We observed a significant number of unfilled SWCNTs with small diameters (**Figure** **2**a). This confirmed that SWCNTs with insufficient diameters (<1.3 nm) cannot accommodate C_70_.^[^ ^43^, ^44^, ^45^, ^46^ ^]^ Figure 2b–d presents typical AC‐TEM images of the C_70_ filled Tuball SWCNTs (C_70_@Tuball), demonstrating a high filling efficiency of C_70_ in SWCNTs with large‐diameters. In Figure 2b, for SWCNTs with diameters of 1.3–1.4 nm, the C_70_ molecules were spatially constrained by nanotube walls and aligned stably along the nanotube axis in a single‐chain configuration, and the minimum observed diameter of SWCNT with single‐chain C_70_ arrangement is 1.32 nm (Figure S1, Supporting Information). As the nanotube diameter increased, we found that the C_70_ molecules transformed from a single‐chain to a double‐chain structure (Figure 2c). In single chains, the spacing between the nanotube wall and C_70_ in this diameter range was measured to be ≈0.33 ± 0.01 nm (Table S1, Supporting Information), close to the interlayer spacing of graphene (0.335 nm). This spacing distance together with the diameter of C_70_ (ca. 0.7 nm) explains that the sufficient nanotube diameter for C_70_ accommodation is larger than 1.3 nm. Raman spectroscopy studies by Iijima's group suggest that, under the equilibrium distance of 0.34 nm, the main interaction between fullerene and SWCNTs is the hybridization of electronic states.^[^ ^43^, ^44^, ^45^, ^46^ ^]^ At the same time, along the nanotube axis, the average spacing for the centroids of neighboring C_70_ molecules is ca.0.95 nm (Table S1, Supporting Information), which is close to the C_70_‐C_70_ distance in their crystals. Whereas, in the same radial direction of SWCNTs, the C_70_‐C_70_ distance in the double chains is compressed, meaning a weaker repulsion force between adjacent C_70_. Interestingly, inside the SWCNTs with diameters from 1.6 to 1.8 nm we observed a transition state between linear and double chains, with C_70_ arranging as a double helix structure, as shown in Figure 2d. Karla S. et al have predicted C_70_ helix structures using molecular dynamics simulation.^[^ ^5^ ^]^ Our experimental results are more complex compared with the simulation. Upon C_70_ filling, the SWCNTs do not provide rigid cylindrical confinement but demonstrate a structural deformation, which is consistent with the results reported by Jamie.^[^ ^47^ ^]^ Within the same individual SWCNT, we observed a variation of SWCNT widths from 1.39 to 1.86 nm in the TEM image. By approximating the ellipse with a short axis of 1.39 nm and a long axis of 1.86 nm into a perfect circle, we calculate the SWCNT's diameter before deformation to be 1.63 nm. The carbon nanotube was widened in the direction where two fullerenes were staggered side by side, causing it to narrow in the perpendicular direction. As a result, the double helix exhibits a rotational periodicity several times longer than theoretical predictions. In each period, one full turn of the helix corresponds to a 360‐degree rotation. It is shown that the C_70_‐SWCNT van der Waals distance keeps always ca. 0.3 nm in all assemblies, suggesting the strongest guest‐host interactions in this distance.^[^ ^45^ ^]^ Figure 2e shows the structural models of C_70_ arrangements within SWCNTs of different diameters. More AC‐TEM images of C_70_@Tuball are shown in Figure S1 (Supporting Information).

### 2.2 Diameter and Metallicity Separation of C_70_@SWCNTs

**Figure 3:**
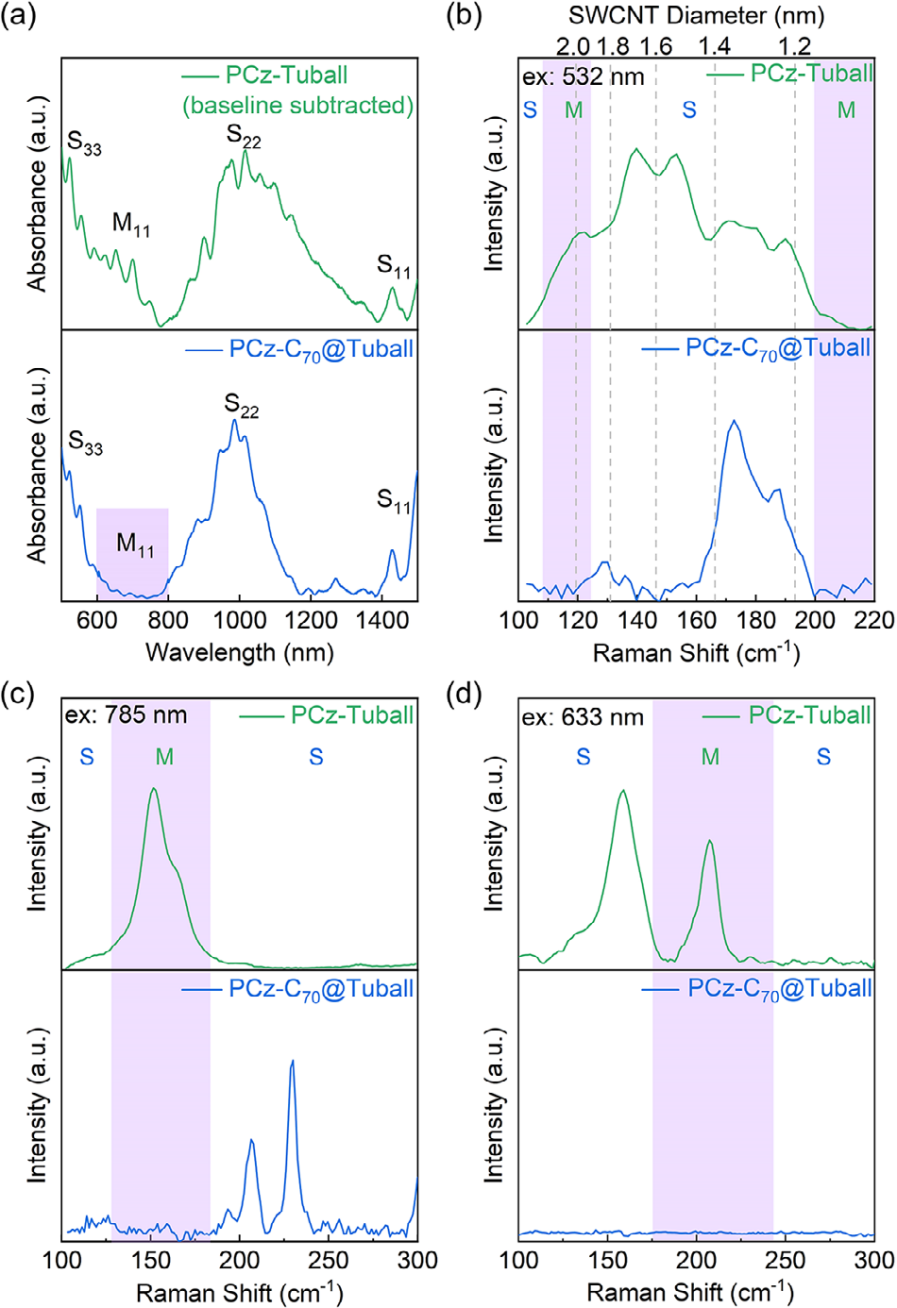
Sorting results using polymer extraction. a) UV–vis–NIR and b–d) Raman spectrum of unfilled and C_70_SWCNTs sorted by PCz polymers. The excitation laser wavelength in Raman is 532, 785, and 633 nm respectively. The Raman shifts are converted to SWCNT diameters.

We find that PCz polymers in toluene can sort C_70_@Tuball with narrowly distributed diameters and semiconducting characteristics. This selectivity is not attainable in unfilled pristine SWCNTs by subjecting the same sorting progress. After the sorting process, PCz solution can disperse unfilled SWCNTs but show selectivity to only C_70_@Tuball samples. **Figure** **3**a presents the ultraviolet−visible−near infrared (UV–vis–NIR) absorption spectrum of supernatants (the full‐range spectrum is shown in Figure S2, Supporting Information). A high baseline background in unfilled SWCNTs due to m‐SWCNTs is subtracted (Figure S3, Supporting Information). The absorption bands located in the 800–1400 nm range correspond to the second van Hove optical transitions (S_22_) of s‐SWCNTs. Those in the 600–800 nm range correspond to primarily the first‐order transitions (M_11_) of m‐SWCNTs, which slightly overlapped with the third van Hove optical transitions (S_33_) of s‐SWCNTs. After PCz sorting, unfilled Tuball SWCNT exhibited broad absorption bands, which corresponds to the broad range of SWCNT diameter (1.2–2.2 nm). In contrast, the S_22_ band in C_70_@SWCNTs significantly narrows toward the absorptions from 900 to 1100 nm of smaller diameter SWCNTs. It indicates that C_70_ encapsulation induced diameter selectivity. s‐SWCNTs with a diameter at 1.3–1.4 nm and S_22_ band at 940–1100 nm possibly assigned to (12,8), (15,5), (13,6), (16,3), (16,2), (11,9), (15,4) species.^[^ ^9^ ^]^ Meanwhile, C_70_@SWCNTs spectrum showed a pronounced downward shift in the M_11_ band, which indicated a sufficient removal of metallic species. This indicated that C_70_ encapsulation can also lead to semiconducting selectivity. To further verify the semiconducting and diameter selectivity of C_70_@SWCNTs after separation, we performed Raman characterizations. Figure 3b shows the radial breathing mode (RBM) regions located in 100–220 cm⁻¹ in the Raman spectra, with a laser excitation wavelength of 532 nm. The RBM of the sorted C_70_@Tuball SWCNTs mainly appeared in the range of 172–190 cm⁻¹. Based on the relationship between nanotube diameter and RBM frequency, *ω *= 222/*d*+8, where *d* represents the diameter of SWCNTs and *ω* represents the RBM frequency, the corresponding nanotube diameter distribution is 1.3–1.4 nm.^[^ ^48^, ^49^ ^]^ This is consistent with the absorption spectra results. Raman measured under 785 nm laser excitation is shown in Figure 3c, also showing the sufficient removal of metallic species. We also observed a minimal amount of narrow‐diameter s‐SWCNTs located ca. 200 cm⁻¹, which should be some residual small‐diameter unfilled SWCNTs. Metallic nanotubes within the 1.3–1.4 nm range are in good resonance with the 633 nm laser, so we also tested the Raman characterization at the excitation wavelength of 633 nm (Figure 3d). RBM peaks corresponding to metallic nanotubes with diameters in the range of 1.3–1.4 nm disappeared significantly, providing further evidence that we have successfully obtained semiconducting SWCNTs. We also tested other sorting polymers and observed a similar diameter selectivity phenomenon after C_70_ filling (Figure S4, Supporting Information). Moreover, we find a similar diameter selectivity in C_70_ filled HiPCO SWCNTs using PCz extraction. (Figure S5, Supporting Information). The consistent results from UV–vis–NIR and Raman spectra confirm the narrow diameter of 1.3–1.4 nm of PCz‐C_70_@SWCNTs.

### 2.3 Host‐Guest Interaction Between C_70_ and SWCNTs

**Figure 4:**
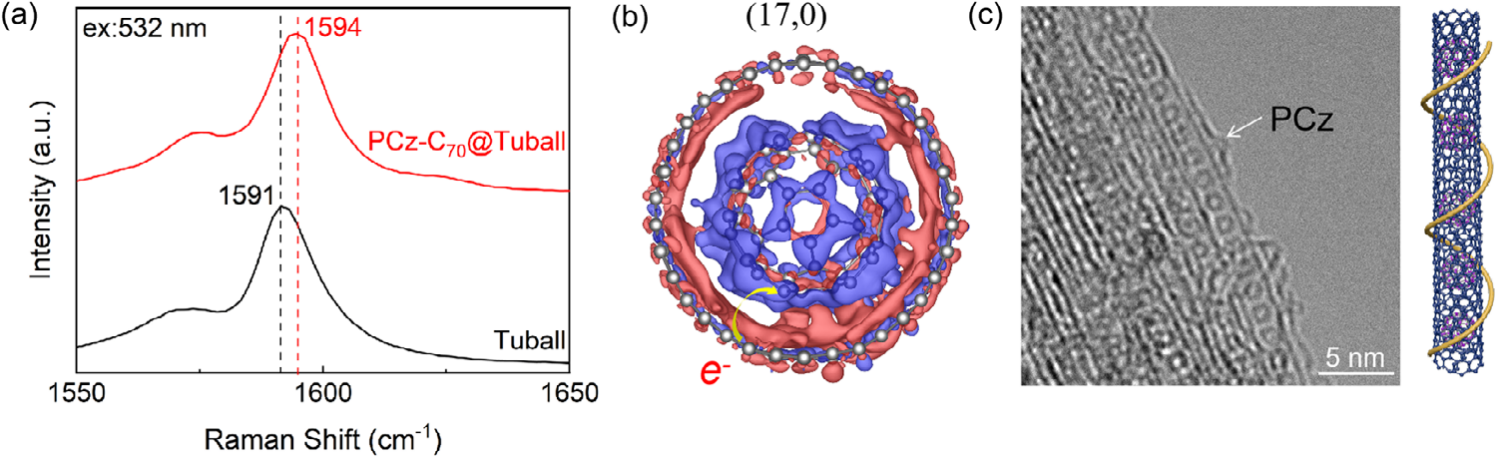
Electrons transfer between SWCNTs and inner C_70_. a) Raman spectra of pristine Tuball and PCz‐C_70_@Tuball film with laser wavelength of 532 nm. The spectra were normalized relative to the intensity of G‐mode. b) DFT calculation showing the differential charge density of C_70_ and (17, 0) SWCNT. The red regions indicate electron depletion, and the blue regions correspond to electron accumulation. c) Representative AC‐TEM image and a structure model of PCz‐C_70_@SWCNTs.

To explore the origin of the selectivity, we perform more characterizations to understand the filling effects of C_70_ on SWCNTs. The close contacts between encapsulated C_70_ and SWCNTs should allow charge redistribution between them. We respectively measured the Raman spectrum of Pristine Tuball and C_70_@Tuball film samples after PCz sorted at different locations to represent the overall situation of the samples, as shown in **Figures** **4**a and S6 (Supporting Information), Raman spectra show the G‐mode of C_70_@Tuball blue shifts of 3 cm⁻¹ with laser wavelength of 532 nm, and 4 cm⁻¹ with laser wavelength of 785 nm from that of unfilled Tuball (Figure S7, Supporting Information), we found that for unfilled Tuball SWCNTs, the Raman G‐mode did not shift before and after PCz separation (Figure S8, Supporting Information). The shift of the G‐mode of Raman spectra can be associated with the movement of the Fermi level of SWCNTs caused by chemical doping. The G‐mode of p‐doped SWCNTs shows an obvious blue shift because of the contraction of the C─C bonds.^[^ ^41^ ^]^ This indicates the SWCNTs donate electrons to the electron acceptor, C_70_. X‐ray photoelectron spectroscopy (XPS) characterization also supports this conclusion as shown in Figure S9 (Supporting Information). The binding energy of C1s for unfilled SWCNTs is 284.8 eV, and shifts by 0.18 eV toward lower energy for C_70_@Tuballs. This indicates the Fermi level downshift of SWCNTs induced by the electron donation to the filled C_70_. We chose s‐SWCNT with a diameter of 13.3Å to encapsulate C_70_ and conducted a density functional theory (DFT) simulation on the charge density of the resultant C_70_@SWCNT (Figure 4b), indicating electrons transfer from the SWCNT to C_70_ in the C_70_@SWCNT heterostructure, thereby inducing a charge density difference—a local positive charging in the SWCNT. This positive charging may alter the wrapping morphology of PCz on SWCNTs, therefore leading to a different sorting result from that of the unfilled, uncharged SWCNTs. The band structures of both C_70_@SWCNT and SWCNT (17,0) are shown in Figure S10 (Supporting Information). The conduction band minimum and the valence band maximum of C_70_@SWCNT are attributed to C_70_ and SWCNT, respectively, indicating a type II heterostructure. Compared to the isolated SWCNT, the electronic states near the valence band of C_70_@SWCNT exhibit an upward shift, resulting in a p‐type doping effect, which is consistent with the charge transfer from SWCNT to C_70_. These results demonstrate a significant modification of the electronic structure of SWCNT due to the encapsulation of C_70_. Early studies from Iijima's group show that, when the distance between the C_70_ and the CNT walls is ca. 0.34 nm, the interaction is strongest, corresponding to nanotubes with a diameter ca.1.4 nm,^[^ ^44^, ^45^ ^]^ close to the diameter of PCz‐C_70_@Tuball. Their studies also show that filled fullerene interacts stronger with s‐SWCNTs than m‐SWCNTs.^[^ ^46^ ^]^ This means that C_70_ filling can enlarge the difference between semiconducting and metallic SWCNTs, facilitating the PCz sorting. All the above suggest that a strong C_70_‐SWCNT intermolecular interaction is a prerequisite for the diameter selectivity of PCz sorting. Figure 4c presents a PCz‐C_70_@SWCNT with ca. 1.35 nm diameter, with C_70_ clearly observed. More AC‐TEM images of PCz‐C_70_@SWCNT are shown in Figure S11 (Supporting Information).

### 2.4 C_70_@s‐SWCNT FETs

**Figure 5:**
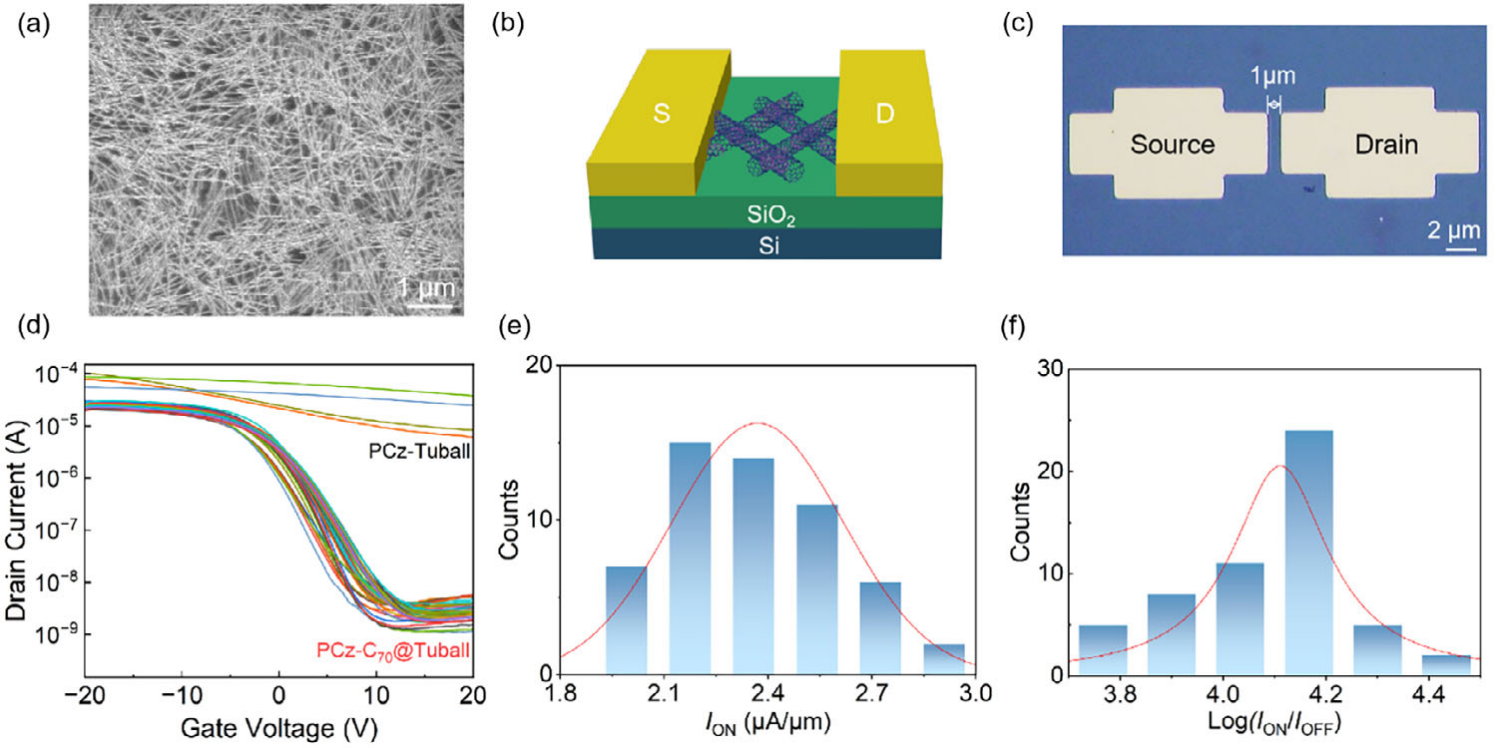
Electrical measurements on PCz‐C_70_@s‐SWCNT FETs. a) SEM image of PCz‐C_70_@Tuball film network. b) Schematic for PCz‐C_70_@Tuball FET device. c) CLSM image of a single PCz‐C_70_@Tuball FET device. d) Transfer curves of the devices. The source−drain bias is −1 V. e) The statistical distribution of on‐state current density. f) The statistical distribution of the on/off ratio.

We fabricated field‐effect transistor (FET) devices by depositing a high‐density random network film of PCz‐C_70_@Tuball on Si substrate with an oxide layer thickness of 100 nm, followed by standard photolithography patterning techniques and source/drain electrodes evaporation, and more details are in the Methods part. **Figure** **5**a shows the SEM image of the prepared film, containing uniform and clean PCz‐C_70_@Tuball. Figure 5b shows a schematic diagram of the FET device. Figure 5c shows the confocal laser scanning microscope (CLSM) image of a single FET device and the channel length is 1 µm. Figure S12 (Supporting Information) shows the output curve of the PCz‐C_70_@Tuball FET device, indicating a well‐formed ohmic contact between SWCNTs and electrodes. We tested both the PCz‐C_70_@Tuball FET and the PCz‐Tuball FET devices, with the transfer characteristics shown in Figure 5d. Because of the presence of a substantial number of metallic tubes, PCz‐Tuball devices exhibit a neglected on/off ratio and larger on‐state current than PCz‐C_70_@Tuball FETs devices. In contrast, the PCz‐C_70_@Tuball FETs demonstrate the on/off ratio of current ≈10^4^, and the on‐state current density of the PCz‐C_70_@Tuball FETs is an average of 2.24 µA µm^−1^ as shown in Figure 5e,f, unequivocally indicating that semiconductor characteristics of the C_70_@Tuball sorted using PCz. Compared with Arc‐SWCNT FETs, the off‐state current of C_70_@Tuball devices remains comparable, indicating good semiconducting characteristics. However, the average on‐state current density of the PCz‐C_70_@Tuball FETs is not quite impressive compared with traditional Arc‐SWCNT FETs at the same channel length. We think the reason why the average on‐state current density is low is possibly because the carrier transport is inhibited to some extent due to the trapping or scattering effect of C_70_. Nevertheless, the C_70_@Tuball FET device still exhibits a switching ratio of 10^4^, which is comparable to Tuball FET devices in the previous report.^[^ ^9^ ^]^ Overall, although C_70_ encapsulation slightly reduces the on‐state current, the resulting devices still retain good semiconducting properties. Our preliminary results suggest that C_70_ filling does not impair the semiconductive characteristics of the carbon nanotubes. Further characterizations, such as studies on potential photo responsiveness will be pursued in future work.

## 3 Conclusion

We developed a precise preparation method for C_70_@SWCNTs heterostructure by combining vapor‐phase filling with after‐filling sorting. For the first time, we selectively extract C_70_@s‐SWCNTs with a narrowly distributed diameter range of 1.3–1.4 nm by PCz polymer. Also, we first observed that C_70_ molecules arrange in a single, double helix, and double chains within the different diameter SWCNTs: in SWCNTs with diameters of 1.3–1.4 nm, C_70_ forms a single chain with a nanotube‐wall distance of ca. 0.33 ± 0.01 nm, close to the graphene interlayer spacing. With tube diameter increasing, we observed a double helix structure in those ≈1.6 nm and a double‐chain structure ca. 1.8 nm. And the double helix exhibits a rotational periodicity several times longer than theoretical predictions, meanwhile causing the deformation of the wall of SWCNT. Shifts in the Raman G‐band, changes in XPS binding energy, and DFT calculations reveal the charge‐transfer interactions between the SWCNTs and fullerene. We conjecture that diameter selectivity should stem from the host‐guest interaction between SWCNTs and C_70_. Using sorted C_70_@s‐SWCNTs, we fabricated thin‐film field‐effect transistor (FET) devices, which exhibit an on/off current ratio ca. 10^4^. This work sheds light on preparing 1‐D molecular assemblies using SWCNT templates.

## 4 Experimental Section

### Preparation of C_70_@SWCNTs

Tuball‐SWCNTs (99% purity) were purchased from OCSiAL company. HiPCO SWCNTs (ca. 40% purity) were purchased from NanoIntegris, and C_70_ (98% purity) was purchased from Sigma–Aldrich. Pristine SWCNTs were annealed in air at 480 °C for 30 min for cap opening. Then, C_70_ and cap‐opened SWCNTs with a weight ratio of 3:1 were sealed in an evacuated quartz tube and heated in a tube furnace. The temperature was raised to 600 °C over 200 min, and held at 600 °C for 7 days. After cooling, the retrieved C_70_@SWCNTs were cleaned several times with toluene, and used bath sonication followed by centrifugation until the supernatant became colorless.

### Polymer Sorting of C_70_@SWCNTs

Poly[9‐(1‐octylonoyl)‐9H‐carbazole‐2,7‐diyl] (PCz) was purchased from Suzhou Xiyin Nanotechnology Co., 10 mg C_70_@Tuball and 20 mg PCz were added to 20 mL toluene. The mixture was sonicated using a probe sonicator (40% output power, 2‐second intervals) for 40 min with water cooling to obtain a stable nanotube dispersion. The dispersion was then ultracentrifuged for 1 h, and the supernatant was collected after centrifugation. Pristine Tuball‐SWCNTs were separated under the same conditions as control experiments.

### Characterization of Filled SWCNTs

Aberration Corrected Transmission Electron Microscope (AC‐TEM) images were acquired from ThermoFisher titan g2 operating at 80KV. Ultraviolet−visible−Near Infrared (UV–vis–NIR) absorption spectrum was collected using a Cary 5000 spectrometer. Raman spectra were acquired using a LabRAM HR‐800 laser confocal Raman spectrometer with laser excitation wavelengths of 532 nm and 785 nm. Scanning Electron Microscope (SEM) images were acquired by a Hitachi S4800.

### DFT Calculations

Density functional theory (DFT) calculations^[^ ^50^, ^51^, ^52^, ^53^ ^]^ were conducted by the Vienna ab initio simulation package (VASP),^[^ ^50^ ^]^ the projector augmented wave (PAW)^[^ ^51^ ^]^ potentials for ion‐electron interaction, and the generalized gradient approximation (GGA) parametrized by Perdew, Burke, and Ernzerhof (PBE) for the exchange‐correction functional.^[^ ^52^ ^]^ The energy cutoff of 400 eV was used for the planewave basis set. The van der Waals interactions between C_70_ and SWCNT (17, 0) were described using Grimme's semiempirical DFT‐D3 dispersion correction scheme.^[^ ^53^ ^]^ A 15 Å vacuum space was incorporated in the vertical direction.

### FET Devices

A high‐density random SWCNT network film of PCz‐C_70_@Tuball on Si substrate with an oxide layer thickness of 100 nm was prepared before device fabrication by deposition for 4 h. Then FET devices with a channel length of 1 µm were fabricated using standard UV photolithography techniques. The source and drain electrodes (Ti /Au, 3/45 nm) layer was deposited using electron beam evaporation. Oxygen plasma etching was used to remove SWCNTs out of the FET channel to prevent electric leakage. CLSM image of the devices was characterized by VX‐250. The device's electronic performance was tested using a Keithley 4200 semiconductor parameter analyzer.

## Conflict of Interest

The authors declare no conflict of interest.

## Author Contributions

Y.H., J.Y., and Y.L. contributed equally. D.Z., S.Q., and L.K. formulated the idea and supervised the experiment. Y.H. prepared the C_70_@SWCNTs heterostructures and separated and characterized the sorted‐SWCNT with the assistance of J.Y., F.J., Y.P., and Z.N. Y.H. and J.Y. performed the device fabrication and properties characterizations. Y.H., S.Q., Y.L., and J.Y. prepared the figures and wrote the manuscript. All authors discussed the results and commented on the manuscript.

## Supporting information



Supporting Information

## Data Availability

The data that support the findings of this study are available from the corresponding author upon reasonable request.
