# Supplementary material for: Selective Sorting of Semiconducting C70@Single‐Walled Carbon Nanotube Heterostructures with Narrow Diameter Distribution
Source: Adv Sci (Weinh). 2025 Mar 16;12(18):2500933. doi: 10.1002/advs.202500933 (PMC12079446; doi:10.1002/advs.202500933)
Supplement: Supplementary file 1 — Supporting Information [file ADVS-12-2500933-s001.docx]

**Supporting Information**

**Selective Sorting of Semiconducting C_70_@Single-walled Carbon Nanotube Heterostructures with Narrow Diameter Distribution**

Yuqi He^#1,2^, Jian Yao^#2,3^, Ye Liu^#2^, Feng Jin^2^, Yujie Peng^2,3^, Zeyuan Nan^2^, Aling Chen^1^, Hehua Jin^2,3^, Song Qiu*^2,3^, Lixing Kang*^2,3^, Dengsong Zhang*^1^, Qingwen Li^2,3^

1. Department of Chemistry, College of Sciences, Shanghai University, Shanghai 200444, P.R. China
2. Advanced Materials Division, Suzhou Institute of Nano-Tech and Nano-Bionics, Chinese Academy of Sciences, Suzhou 215123, P.R. China
3. School of Nano-Technology and Nano-Bionics, University of Science and Technology of China, Hefei 230026, P.R. China

**Table S1.** Average values of d_1_ and d_2_ in C_70_@SWCNTs of single chain structure

**
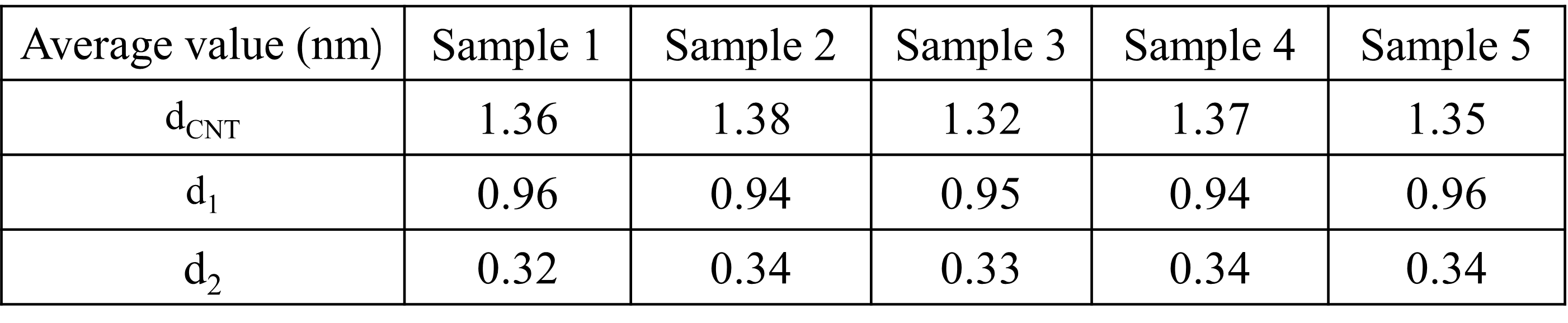
**


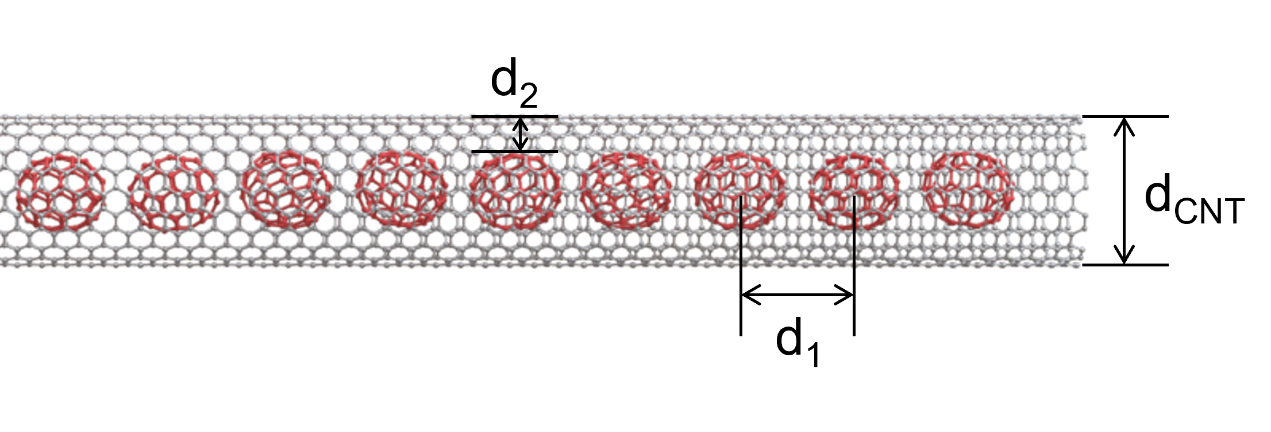
Note: *d_CNT_* is the diameter of SWCNTs, *d_1_* is the distance between the centroids of adjacent C_70_ molecules, *d_2_* is the distant between wall of SWCNTs and inner C_70_.

We analyzed average values of *d_1_* and *d_2_* in C_70_@SWCNTs of single chain structure based on the AC-TEM images, the distance between the nanotube wall and C_70_ in this diameter range was measured to be approximate 0.33 ± 0.01 nm (**Table S1**), closed to the interlayer spacing of graphene (0.335 nm). Under the equilibrium distance of 0.34 nm, the main interaction between fullerene and SWCNTs is the hybridization of electronic states.^1–3^ In the same time, along the nanotube axis, the average distance between the centroids of adjacent C_70_ molecules is about 0.95 nm (**Table S1**), which is close to the C_70_-C_70_ distance in their crystals.


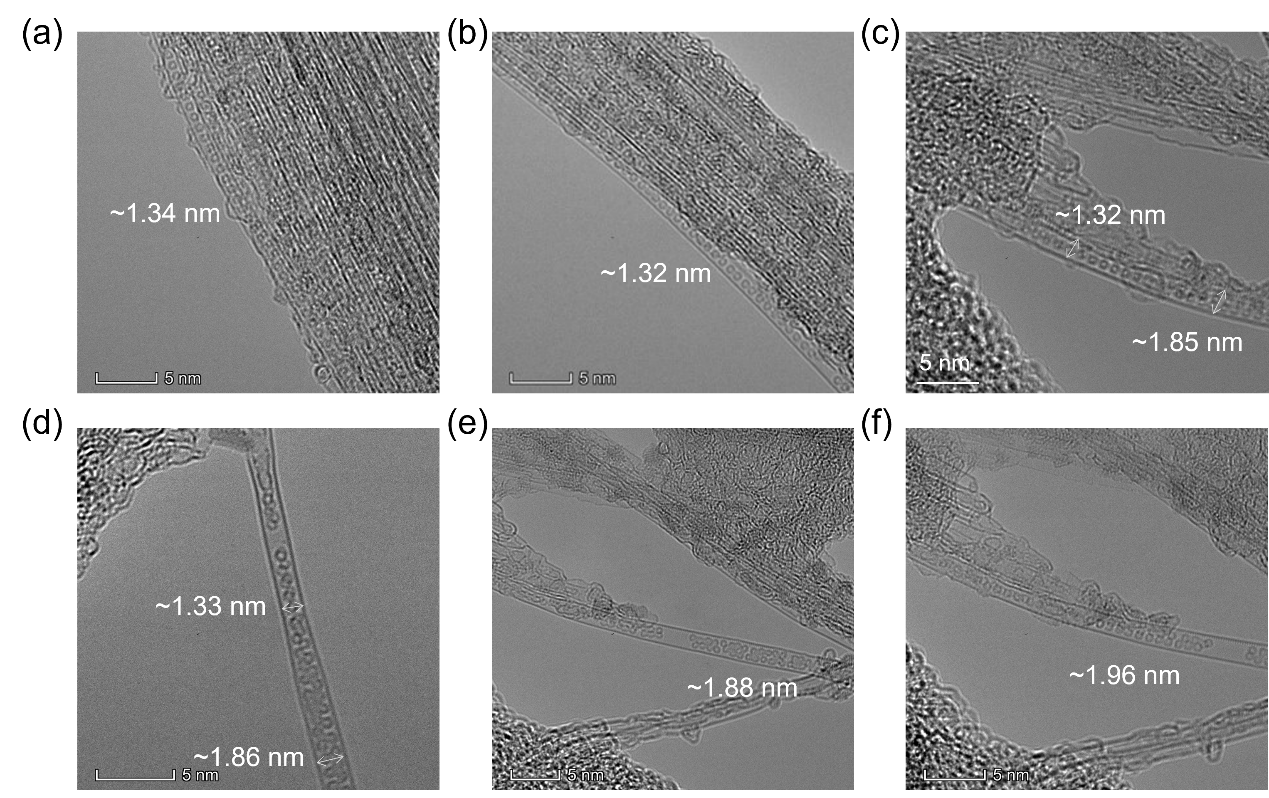


**Figure S1.** AC-TEM images of C_70_@Tuball showing different ordering configurations of encapsulated C_70_. (a) and (b): single chain, (c) and (d): double helix structure, (e) and (f): double chain.


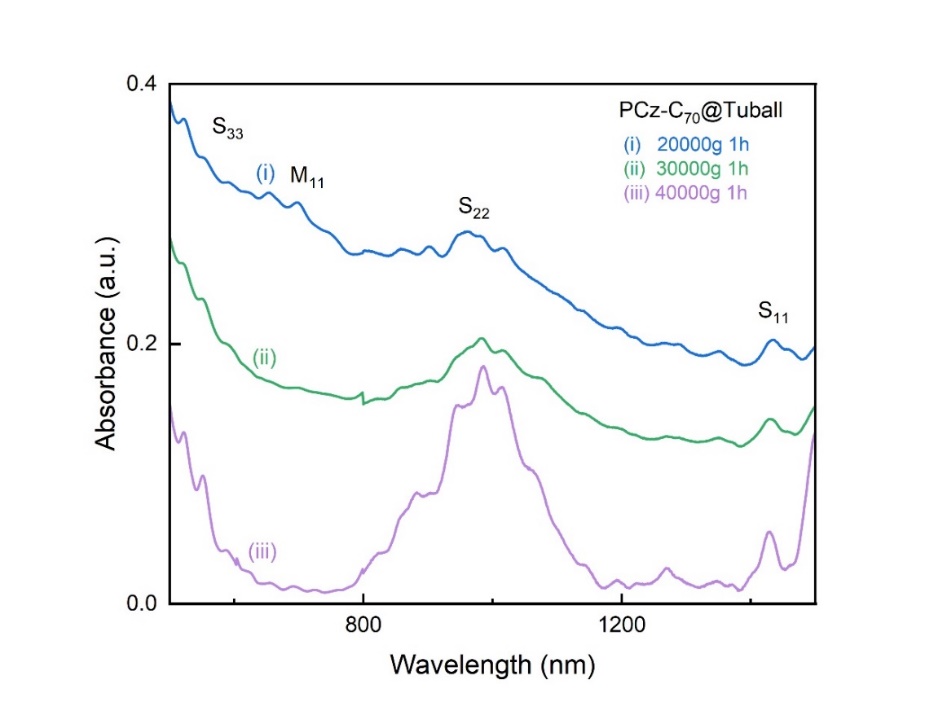


**Figure S2.** UV-vis-NIR absorption spectra of PCz sorted C_70_@SWCNTs. Centrifugal force: 20000, 30000, 40000g. The centrifugation time is maintained at 1 h.


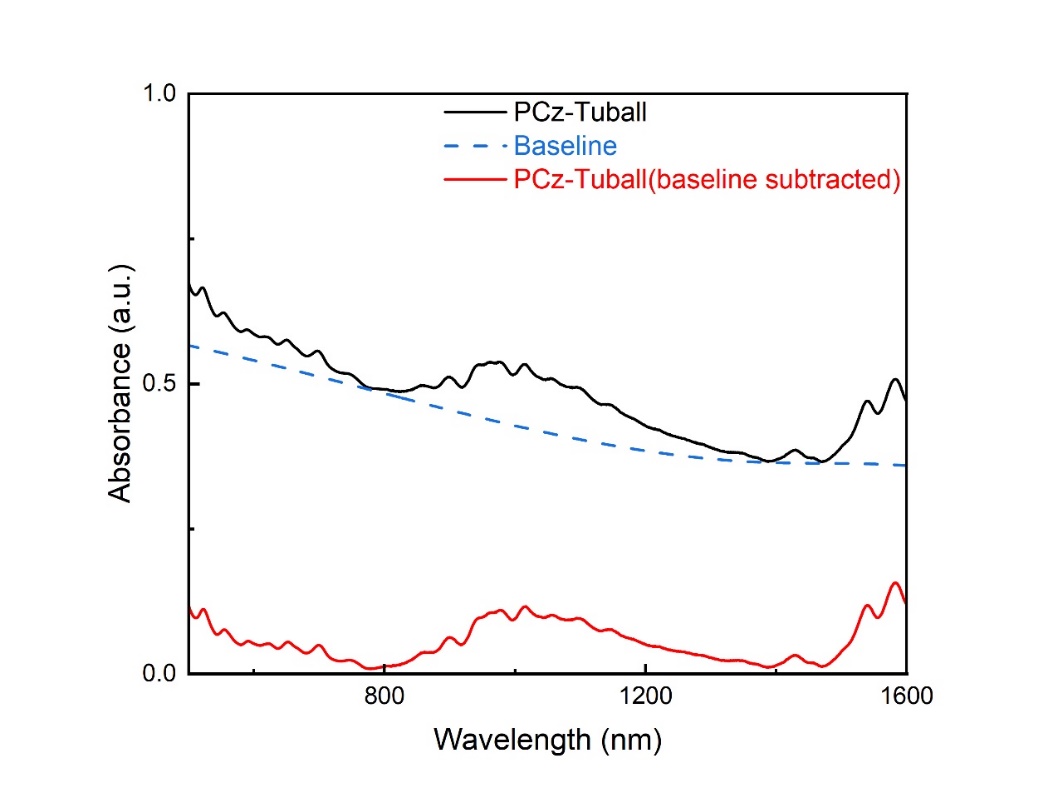


**Figure S3.** Baseline subtracted in optical spectra of PCz sorted Pristine Tuball.


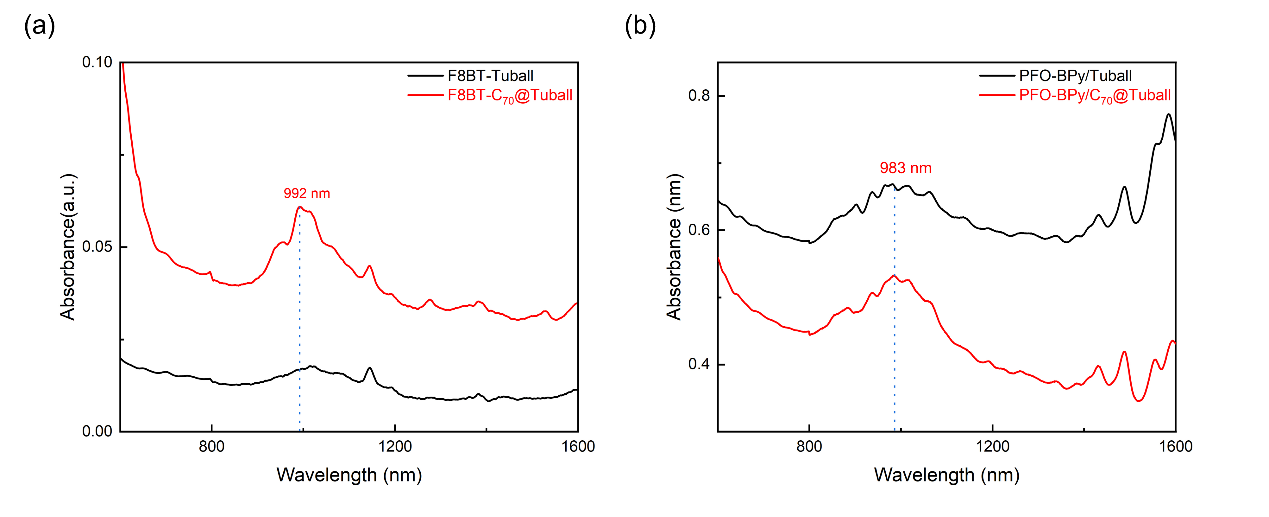


**Figure S4.** UV-vis-NIR absorption spectra of (a) F8BT sorted Pristine Tuball and C_70_@Tuball. (b) PFO-BPy sorted Pristine Tuball and C_70_@Tuball.


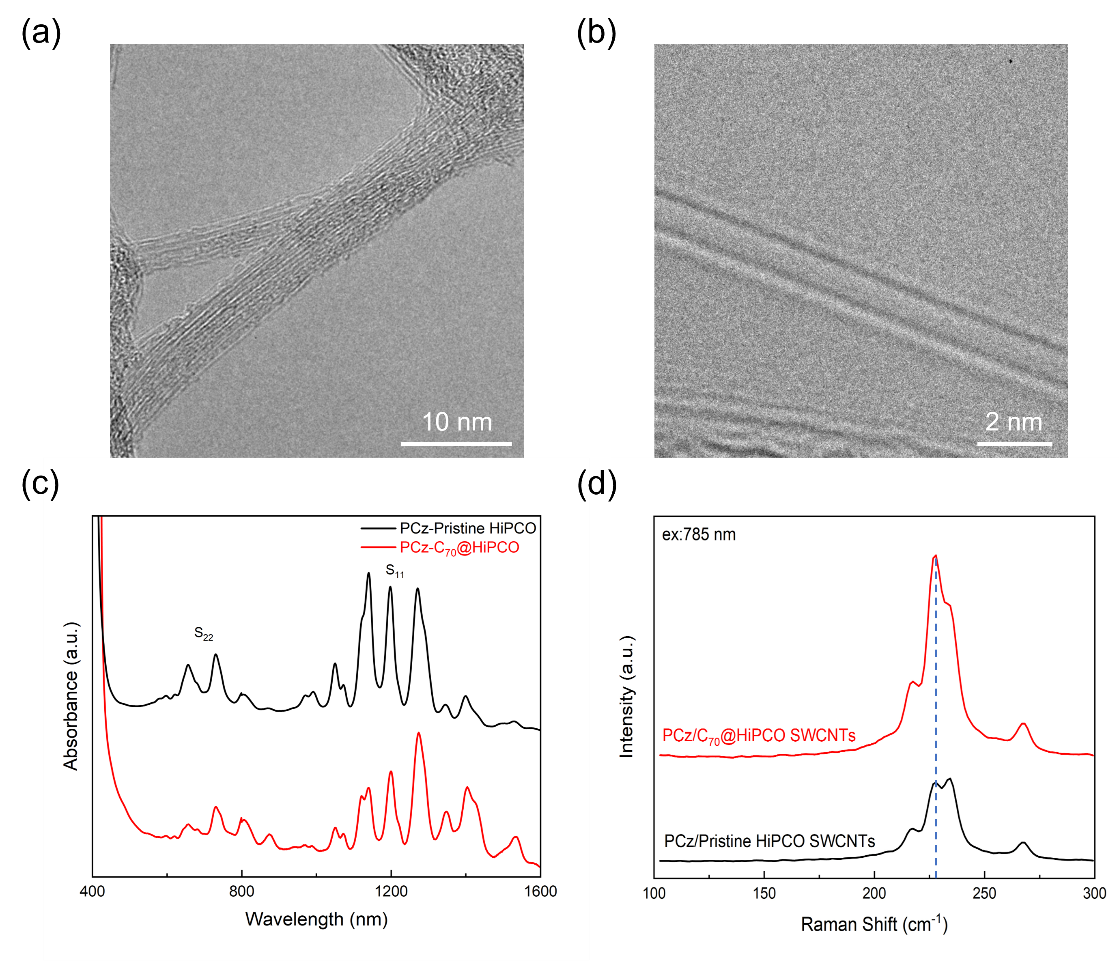


**Figure S5.** Encapsulation and sorting results of C_70_@HiPCO using polymer extraction. (**a-b**). AC-TEM images of HiPCO SWCNTs after C_70_ filling. (**c**) UV-vis-NIR and (**d**) Raman spectra of PCz-HiPCO and PCz-C_70_@HiPCO. The Raman signals are collected under a 785 nm laser excitation.

In the C_70_ encapsulation experiments, we also filled small-diameter HiPCO SWCNTs (0.8 to 1.2 nm). As shown in **Figure S5-a and b**, we observed a significant number of unfilled SWCNTs in the HiPCO samples by AC-TEM, indicating insufficient diameters to accommodate C_70_ molecules. We then compared the UV-Vis-NIR absorption spectra before and after C_70_ filling. **Figure S5-c** shows the UV-vis-NIR absorption spectra of pristine HiPCO and C_70_@HiPCO sorted by PCz. Because there are a large number of small-diameter unfilled SWCNT in the dispersion, the obtained absorption spectrum represents a mixture of filled and unfilled samples. The absorption bands range from 1000 to 1400 nm correspond to the first van Hove optical transitions for s-SWCNTs absorption band (S_11_), and those in the 600-1000 nm range correspond to the second van Hove optical transitions for s-SWCNTs absorption band (S_22_). We observed the proportion of different chiral SWCNTs sorted by PCz changes after C_70_ encapsulation from the spectra, with a significant absorbance increase where wavelength range from 1270 to 1600 nm, indicating a certain degree of enrichment of larger-diameter SWCNTs filled by C_70_. Raman spectroscopy indicates an enhancement of the RBM signal near 230 cm⁻¹ under 785 nm laser excitation (**Figure S5-d**), suggesting a certain degree of enrichment of large-diameter SWCNTs. This result is consistent with the results characterized by the absorption spectra.


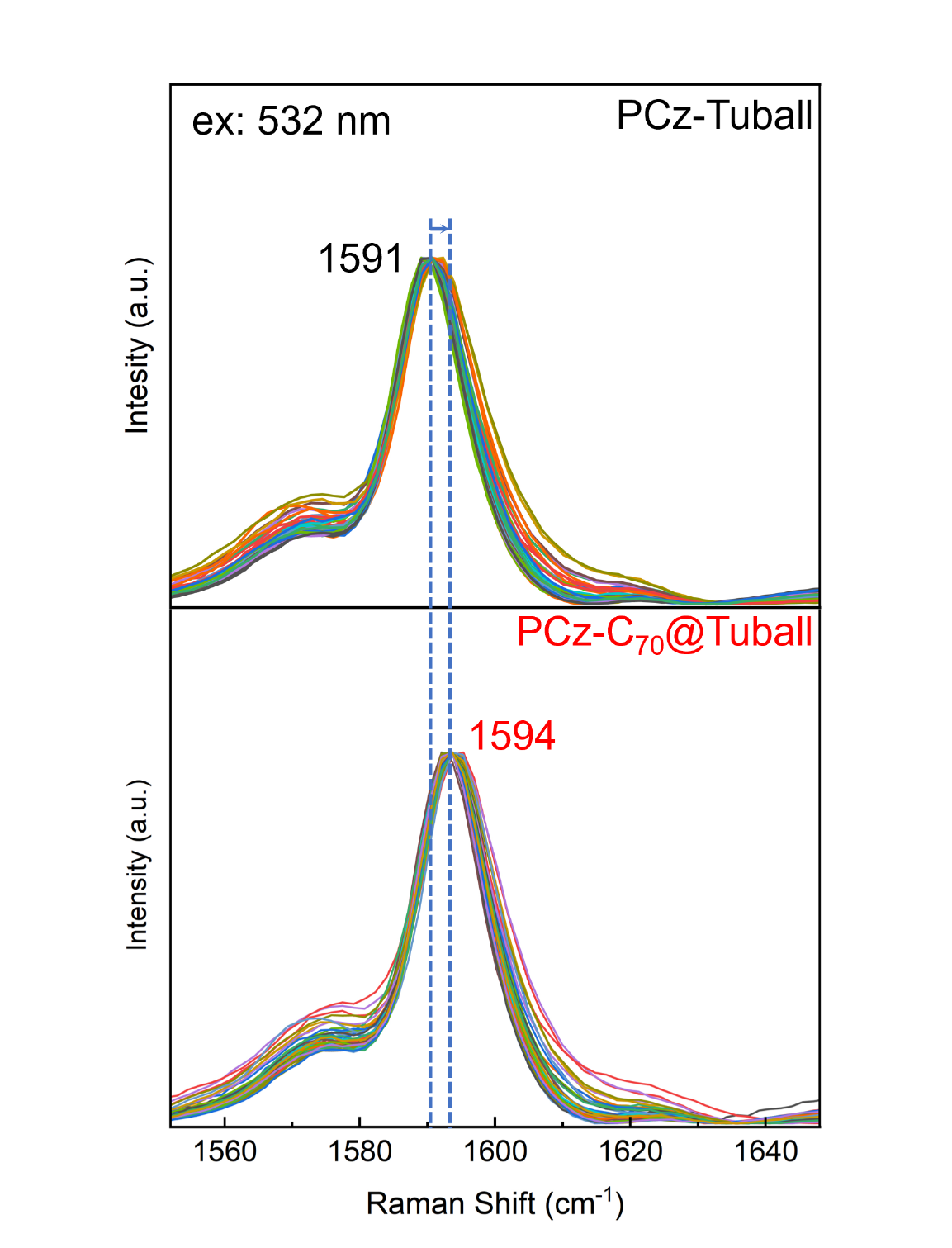


**Figure S6.** Raman spectra of pristine Tuball and PCz-C_70_@Tuball with laser wavelength of 532 nm. The spectra were normalized with respect to G-peak intensity.


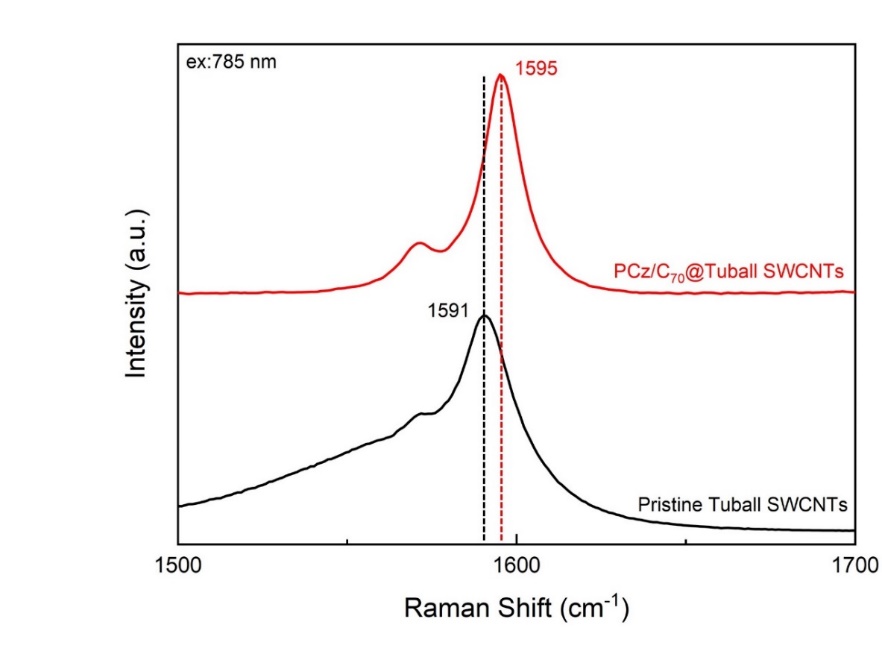


**Figure S7.** Raman spectra of pristine Tuball and PCz-C_70_@Tuball with laser wavelength of 785 nm. The spectra were normalized with respect to G-peak intensity.


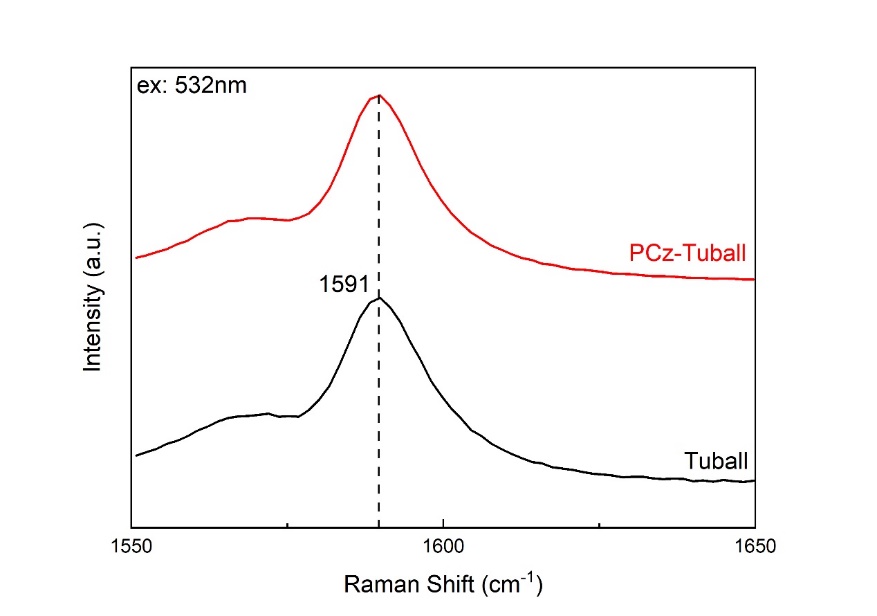


**Figure S8.** Raman spectra of Tuball and PCz-Tuball with laser wavelength of 785 nm. The spectra were normalized with respect to G-peak intensity.


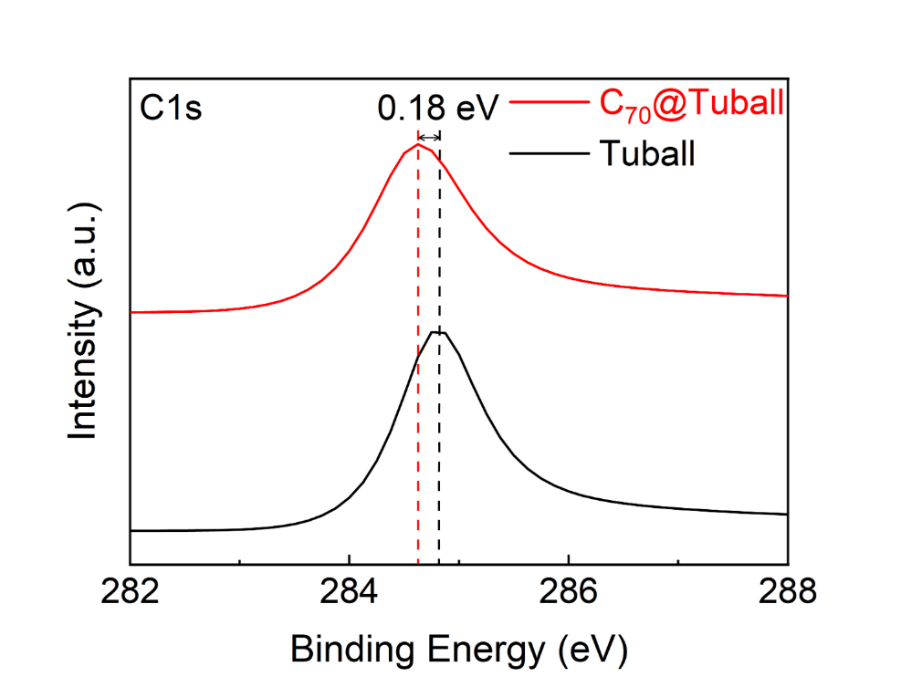


**Figure S9.** XPS spectra of pristine Tuball and C_70_@Tuball in the C1s.


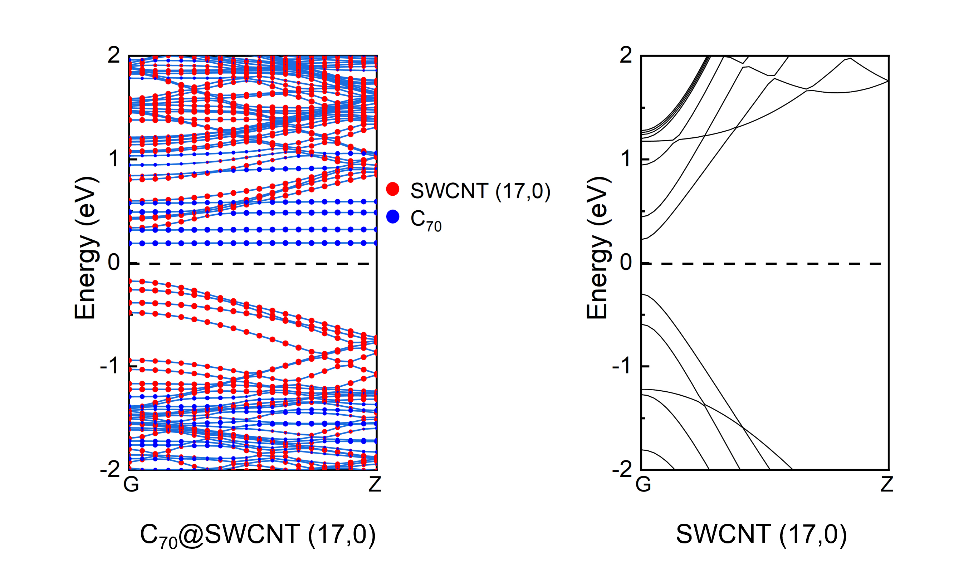


**Figure S10.** DFT calculation showing the energy band structures of C_70_@SWCNT (17, 0) and SWCNT (17,0).


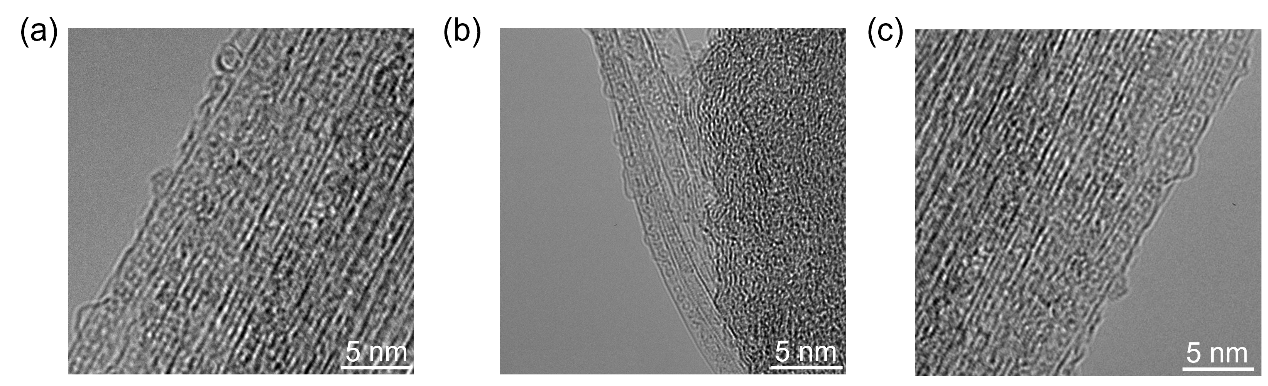


**Figure S11.** AC-TEM images of PCz-C_70_@SWCNT.

\


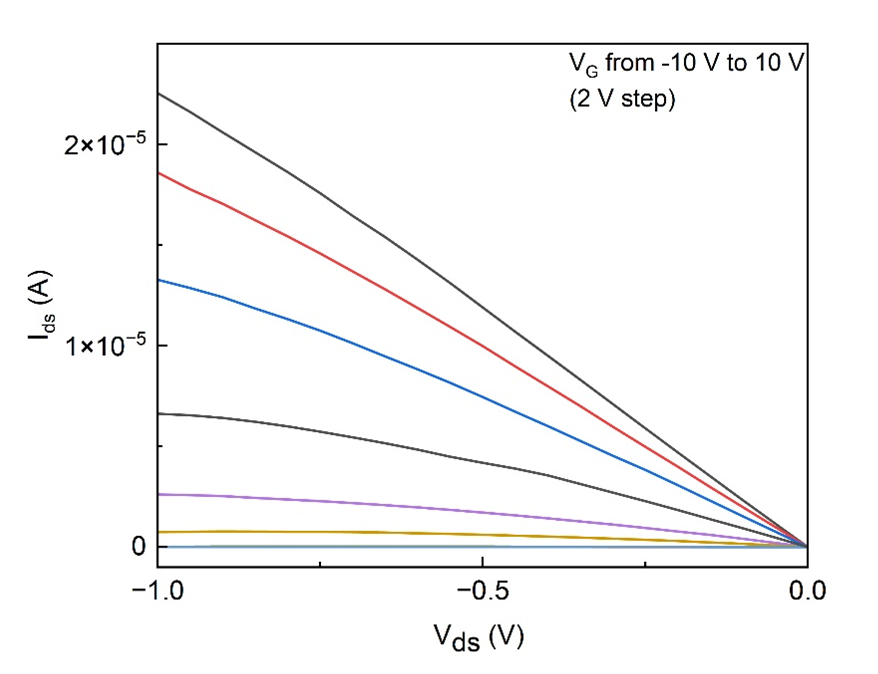


**Figure S12.** The output curve of PCz-C_70_@Tuball FET device.

Reference

1. Joung, S.-K.; Okazaki, T.; Okada, S.; Iijima, S. Interaction between Single-Wall Carbon Nanotubes and Encapsulated C_60_ Probed by Resonance Raman Spectroscopy. *Phys. Chem. Chem. Phys.* **2010**, *12* (28), 8118.
2. Joung, S.; Okazaki, T.; Okada, S.; Iijima, S. Host–Guest Interaction between Single‐wall Carbon Nanotubes and Encapsulated C_60_ Probed by Resonance Raman Spectroscopy. *Phys. Status Solidi (b).* **2010**, *247* (11–12), 2700–2702.
3. Joung, S.-K.; Okazaki, T.; Okada, S.; Iijima, S. Weak Response of Metallic Single-Walled Carbon Nanotubes to C_60_ Encapsulation Studied by Resonance Raman Spectroscopy. *J. Phys. Chem. C* **2012**, *116* (44), 23844–23850.
